# Zenker's diverticulum treated with submucosal tunneling endoscopic septum division surgery: Two case reports

**DOI:** 10.1097/MD.0000000000018659

**Published:** 2020-01-31

**Authors:** Baile Shen, Haizhong Jiang, Zhuoya Li, Zheng Wang, Haojun Song, Xiaoyun Ding

**Affiliations:** a Medical School of Ningbo University; b Department of Gastroenterology, Ningbo First Hospital, Ningbo; c Medical School of Zhejiang University, Hangzhou, Zhejiang Province, China.

**Keywords:** dysphagia, submucosal tunneling endoscopic septum division, Zenker's diverticulum

## Abstract

**Introduction:**

Zenker's diverticulum (ZD) refers to a pouch-like structure similar to the esophageal lumen formed from the herniation of the esophageal mucosa; this structure makes it difficult for food to pass through the esophagus to the stomach. The development of endoscopic technology has made minimally invasive surgical treatments for ZD possible.

**Patient concerns:**

A female 72-year-old patient was admitted to our hospital due to recurrent dysphagia for more than 5 years. A 62-year-old female patient underwent a gastroscopic examination due to recurrent dysphagia for 10 years and aggravated dysphagia accompanied by bad breath for 1 year.

**Diagnosis:**

A significant diverticulum with food residue at the entrance of the esophagus was found on gastroscopy in both cases.

**Interventions:**

After completing a relevant examination and excluding surgical contraindications, both patients underwent submucosal tunneling endoscopic septum division.

**Outcomes:**

Both patients were discharged after symptoms alleviated on postoperative day 4. A 3-month follow-up gastroscopy showed the disappearance of the diverticulum and recovery of the esophageal anatomical structure. No symptom relapse was found at the 6-month follow-up assessment.

**Conclusion:**

Submucosal tunneling endoscopic septum division has become the most common minimally invasive treatment option. It is efficient and safe for relieving symptomatic ZD in the short term.

## 1 Introduction

Zenker's diverticulum (ZD), also known as pharyngoesophageal diverticulum, is a special type of esophageal diverticulum that often manifests as bulging of the weak region between the inferior pharyngeal constrictor muscle and the cricopharyngeal muscle.^[1]^ ZD primarily affects older individuals and is easily misdiagnosed as thyroid tumor given its location posterior to the thyroid.^[2]^ Patients with ZD are normally diagnosed on gastroscopy because of symptoms such as dysphagia and reflux. Previously, ZD was mostly treated with surgery; however, the development of endoscopic technology has made the treatment of esophageal diverticulum under endoscope possible. Here, we report two cases of ZD treated using submucosal tunneling endoscopic septum division (STESD).

## 2 Case report

### 2.1 Case 1

**Figure 1:**
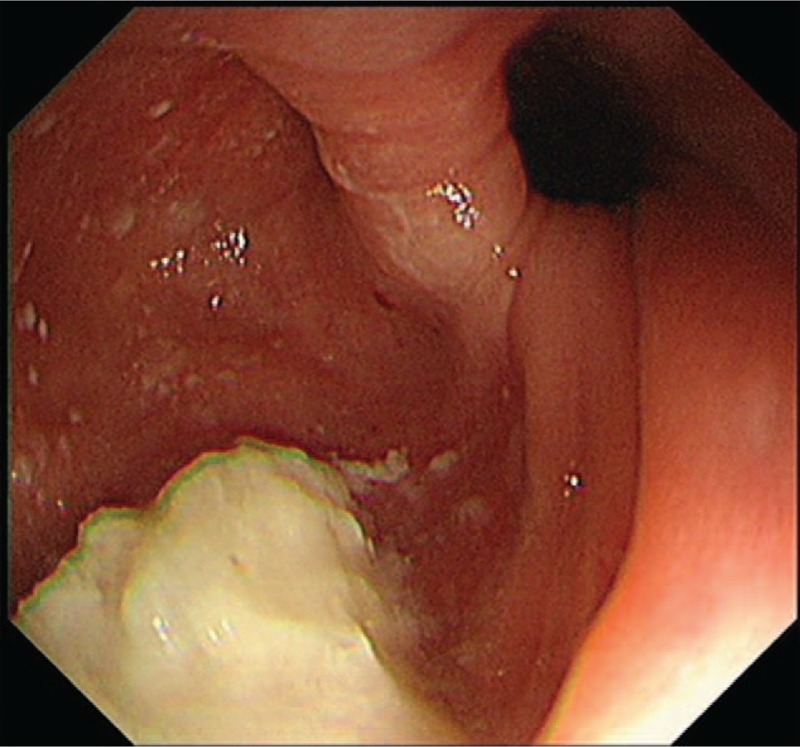
A large diverticulum was identified on gastroscopy at the entry of the esophagus.

A 72-year-old female patient was admitted to our hospital due to recurrent dysphagia for more than 5 years and aggravated dysphagia for 1 year. Two years prior to admission, the patient started to experience dysphagia but had no symptoms of hematemesis, melena, diarrhea, abdominal pain, headache, dizziness, progressive dysphagia, or history of special diseases. A physical examination showed no abnormal heart–lung auscultation, a flat belly, a soft abdominal wall, no palpation or rebound tenderness, and no involvement of the liver, spleen, or under the ribs. A routine blood examination showed that the white blood cell count (WBC) was 5.39 (∗10^9/L), heartbeat (HB) was 112 (g/L), and platelet count (PLT) was 243 (∗10^9/L), and the fecal occult blood test was weakly positive. A significant diverticulum with food residue was found on gastroscopy at the entrance of the esophagus (Fig. 1). The patient was diagnosed with ZD.

**Figure 2:**
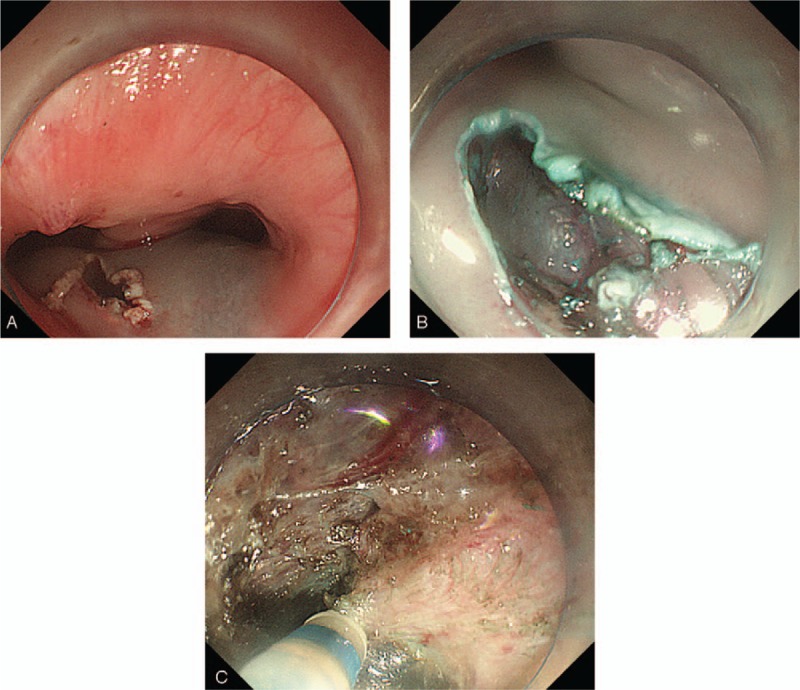
Treatment of ZD using STESD. (A) Adrenaline saline was injected submucosally 3 cm above the diverticular septum, and a longitudinal mucosal incision was made with a hook knife. (B) A submucosal tunnel to the diverticular septum was created. (C) The muscle layer of the septum was dissected down to the bottom of the diverticulum with a high frequency electric coagulation scalpel.

Because of her age and the invasiveness of surgical excision, she opted for treatment with STESD. After completing a relevant examination and excluding surgical contraindications, the patient signed an informed consent document and underwent STESD under general anesthesia. A diverticulum was found at the esophageal entry. Adrenaline saline was injected submucosally at 3 cm above the diverticular septum. A longitudinal mucosal incision was made using a hook knife. A submucosal tunnel was established to the diverticular septum. The mucosal layer at both sides of the septum was isolated and fully exposed. Then, the muscle layer was dissected from the middle of the septum down to the bottom of the diverticulum using a Dual knife. The wound was treated with coagulation forceps, and the tunnel entry was closed with titanium clips (Fig. 2). Any mucosal injury or perforation should also be clipped. The operation was completed in <1 h. Minor bleeding occurred after septotomy and stopped after endoscopic placement of endoclips. The patient fasted without food or water intake, and intravenous antibiotics and nutrition rehydration were given for 2 days postsurgery. Semi-fluid diet was resumed on postoperative day 3, and the patient was discharged after the symptoms alleviated on postoperative day 4. A 3-month follow-up gastroscopy showed the disappearance of the diverticulum and the recovery of the esophageal anatomical structure. No symptom relapse was found at the 6-month follow-up assessment.

### 2.2 Case 2

A 62-year-old female patient underwent a gastroscopic examination due to “recurrent dysphagia for 10 years and aggravated dysphagia accompanied by bad breath for 1 year.” A diverticulum with a diameter of 2.5 cm and depth of 3.0 cm was found below the posterior wall of the esophageal entry, 15 cm from the incisors. A 1.5-cm-long ridge-like tissue was found between the diverticulum and the esophageal lumen. Food residue and fluid were found inside the diverticulum. The patient was diagnosed with ZD. The patient was mostly healthy and did not have abdominal pain, diarrhea, heartburn, reflux, headache, or dizziness. A physical examination showed no abnormal heart–lung auscultation, a flat belly, soft abdominal wall, no palpation or rebound tenderness, and no involvement of the liver, spleen, or under the ribs. After completing the relevant examination and signing an informed consent document, the patient underwent STESD under general anesthesia. Adrenaline saline was injected submucosally 3 cm above the right side of the diverticular septum. A 2.0-cm longitudinal mucosal incision was made with a hook knife, and a tunnel was established. The mucosal layer was separated using a Dual knife to the muscle layer of the diverticular septum. Then, the muscle layer was dissected from the middle of the septum down to the bottom of the diverticulum. The wound was treated with coagulation forceps, and the mucosal incision was closed with titanium clips. The procedure time was 45 min, and minor bleeding occurred during the operation. The patient fasted without food or water intake, and intravenous antibiotics and nutrition rehydration were given for 3 days after surgery. The patient was discharged after the symptoms alleviated on postoperative day 4. The 3-month follow-up endoscopy showed the disappearance of the diverticulum. No symptom relapse was found at the 6-month follow-up assessment.

## 3 Discussion

ZD is a clinically rare, benign condition of the esophagus that often occurs at the posterior wall of the pharyngoesophageal junction. Currently, the etiology of ZD remains unclear. Most researchers believe that ZD occurs because of a weak small region called the Killian triangle, which is formed by lack of muscle fibers at the junction between the inferior pharyngeal constrictor oblique muscle and the cricopharyngeus transverse muscle.^[1]^ Under increased esophageal pressure, the mucosal and submucosal layers balloon out, forming a pouch-like structure. Because the weak region is mostly localized on the left side, ZD is often found on the left side of the neck.^[3]^ ZD progresses slowly, and the disease can last for several years. The most common symptom of ZD is dysphagia. In addition, ZD can present with symptoms such as the regurgitation of undigested food. Aspiration of material from the diverticulum into the trachea can lead to lung infection.^[4]^ Occasionally, ZD is accompanied by esophageal ulcers and perforation.

Currently, the diagnosis of ZD primarily relies on the barium swallow test or an endoscopy examination. Multiple studies have also reported the diagnostic value of ultrasound examination for ZD.^[5,6]^ However, ZD should be distinguished from a thyroid nodule to avoid misdiagnosis on ultrasound examination.^[7]^ Because ZD is a rare condition, its surgical treatments are rarely reported.

STESD, a novel branch of submucosal endoscopy, employs a submucosal tunnel as the operating space. Li et al^[8]^ first reported ZD treatment using STESD. They found that STESD is a simple, minimally invasive method that resolves the dysphagia caused by large esophageal diverticula and avoids the significant lesions and slow recovery that accompanies conventional surgery. Despite recent advances and maturation of the newly developed submucosal tunneling endoscopic technique, there are very few literature reports of this technique in the treatment of ZD. In many areas, the conventional endoscopic procedure is still used for ZD, and it is thus necessary to conduct further clinical studies to confirm its curative effect.

ZD is generally prevalent in old-aged patients, who are usually over 55 years old and are burdened with symptoms for varied time period (from weeks to years). In this research, the two female patients (mean age, 67) complained of dysphagia for over 5 years and demonstrated aggravating symptoms over the last year. Endoscopy showed a ZD with 15 cm-in length from the incisors. Therefore, STESD was performed on two patients under general anesthesia upon following appropriate preoperative preparation, which successfully treated ZD without any procedure-associated adverse events. The main advantage of STESD over traditional surgical intervention is the maintenance of mucosal integrity in the selective division of the muscular septum, thereby attenuating the risk of postoperative leakage as well as secondary infection. Therefore, patients can be discharged after the symptoms are alleviated in a short time. STESD is a simple, minimally invasive method, and the mean operative time and bleeding loss are undoubtedly reduced compared with conventional surgery. The complete cut-off of the muscle layer from the esophageal wall to diverticulum is considered as the second advantage of STESD. However, the incomplete dissection of septum in the traditional endoscopic treatment could lead to residual diverticulum, residual symptoms or recurrence. The dysphagia of both patients was alleviated in the short term. Recurrence was not found at a 6-month follow-up assessment. An endoscopic examination showed the disappearance of the diverticular septum and the recovery of the esophageal anatomical structure. Nevertheless, further large-scale clinical studies are warranted to validate the speculation.

During the diagnosis of patients with dysphagia, digestive endoscopists should consider the possibility of ZD if they encounter difficulty or abnormal resistance during the insertion of the endoscope and have excluded space-occupying lesions in the upper esophageal segment. Endoscopists should not violently insert an endoscope to avoid perforation and bleeding. Serious complications can be avoided by injecting air in the esophageal entry to maintain clear vision. In clinical practice, surgery using an endoscope has become a more appropriate choice for treating patients with ZD.

## Author contributions

**Data curation:** Zhuoya Li.

**Formal analysis:** Haizhong Jiang, Zhuoya Li.

**Investigation:** Zheng Wang.

**Resources:** Haizhong Jiang.

**Writing – original draft:** Baile Shen.

**Writing – review & editing:** Baile Shen, Haizhong Jiang, Haojun Song, Xiaoyun Ding.
